# Characterization of *Streptococcus pyogenes* from Animal Clinical Specimens, Spain 

**DOI:** 10.3201/eid2312.151146

**Published:** 2017-12

**Authors:** Ana Isabel Vela, Pilar Villalón, Juan Antonio Sáez-Nieto, Gema Chacón, Lucas Domínguez, José Francisco Fernández-Garayzábal

**Affiliations:** Complutense University, Madrid, Spain (A.I. Vela, L. Domínguez, J.F. Fernández-Garayzábal);; Instituto de Salud Carlos III, Majadahonda, Madrid (P. Villalón, J.A. Sáez-Nieto);; Laboratorio Exopol San Mateo, Zaragoza, Spain (G. Chacón)

**Keywords:** Streptococcus pyogenes, animals, characterization, detection, bacteria, Spain, group A Streptococcus, streptococci, erythromycin, antimicrobial resistance, virulence, genes

## Abstract

*Streptococcus pyogenes* appears to be almost exclusively restricted to humans, with few reports on isolation from animals. We provide a detailed characterization (*emm* typing, pulsed-field gel electrophoresis [PFGE], and multilocus sequence typing [MLST]) of 15 *S. pyogenes* isolates from animals associated with different clinical backgrounds. We also investigated erythromycin resistance mechanisms and phenotypes and virulence genes. We observed 2 *emm* types: *emm*12 (11 isolates) and *emm*77 (4 isolates). Similarly, we observed 2 genetic linages, sequence type (ST) 26 and ST63. Most isolates exhibited the M macrolide resistance phenotype and the *mef*A/*erm*B genotype. Isolates were grouped into 2 clones on the basis of *emm*-MLST-PFGE-virulence gene profile combinations: clone 1, characterized by the combined genotype *emm*12-ST36-pulsotype A-s*pe*G; and clone 2, characterized by the genotype *emm*77-ST63-pulsotype B-*spe*C. Our results do not show conclusively that animals may represent a new reservoir of *S. pyogenes* but indicate the ability of human-derived *S. pyogenes* isolates to colonize and infect animals.

*Streptococcus pyogenes* (group A *Streptococcus*) is a gram-positive bacterium that causes several diseases in humans. *S. pyogenes* usually colonizes the throat or skin epithelial surfaces and causes a wide variety of clinical manifestations, such as noninvasive pharyngitis, dermatitis, and scarlet fever (*1**,**2*). However, this pathogen is also responsible for deadly invasive systemic infections such as necrotizing fasciitis and streptococcal toxic shock syndrome (*3*).The ecologic niche of *S. pyogenes* appears to be quite narrow, with humans being the almost exclusive biologic host (*4*) and no animal or environmental reservoir of known importance contributing to its life cycle (*2*). Reports of isolation of *S. pyogenes* from sources other than humans are rare. *S. pyogenes* has recently been associated with an infection in a free-living European hedgehog (*Erinaceus europaeus*) (*5*). *S. pyogenes* has also been recovered from the feces of a dog with possible antibiotic-associated colitis (*6*) and from the eye discharge of a dog with conjunctivitis (*7*). We know of no other reports of isolation of this microorganism from animals.

We conducted a study to provide a detailed characterization of animal *S. pyogenes* isolates using *emm* typing, pulsed-field gel electrophoresis (PFGE), and multilocus sequence typing (MLST). We also investigated erythromycin resistance mechanisms and phenotypes, as well as virulence genes.

## Materials and Methods

### Origin and Identification of Bacterial Isolates

We analyzed 15 isolates of *S. pyogenes* obtained from rabbits (n = 14) and sheep (n = 1) in Spain during 2006–2014 (Table 1). Most rabbit isolates were from unrelated animals, located in different commercial farms (n = 14) and locations throughout Spain. Links between rabbit farms were not identified. The sheep included in this study was from a farm that had no rabbits. Human contact with animals was restricted to the personnel working in the rabbit farms and sheep flocks. 

**Table 1:** Features and disease manifestations of 15 animals from which *Streptococcus pyogenes* isolates were collected, Spain, 2006–2014

Isolate*	Animal	Clinical background	Specimen	Geographic region	Isolation date†
M50163	Rabbit	Metritis	Uterus	Valencia	2006 Jan
M79144	Rabbit	Abscesses and dermatitis	Skin	Valladolid	2013 Mar
M78761	Rabbit	Dermatitis	Skin	Valladolid	2013 Feb
M75791	Rabbit	Abscesses	Skin	Valencia	2012 Apr
M75539	Sheep	Abscesses	Skin	Zaragoza	2012 Mar
M75533	Rabbit	Otitis	Ear	Valencia	2012 Mar
M75123	Rabbit	Metritis	Uterus	Castellón	2012 Feb
M73512	Rabbit	Abortion	Uterus	Zaragoza	2011 Aug
M72636	Rabbit	Metritis	Uterus	Zaragoza	2011 May
M72193	Rabbit	Abscesses	Skin	Valencia	2011 Apr
83639	Rabbit	Abscesses and dermatitis	Skin	Valladolid	2014 Mar
83553	Rabbit	Pneumonia	Lung	Zaragoza	2014 Mar
M82209	Rabbit	Abscesses	Skin	Valladolid	2013 Dec
M75768	Rabbit	Mastitis	Milk	Zaragoza	2012 Mar
85374	Rabbit	Skin infection	Skin	Valladolid	2014 Aug

*Isolates M50163 and M73512 were recovered in pure culture. The remaining isolates were recovered together with *Staphylococcus aureus*. †Except for isolates M79144 and M78761, which were isolated in the same farm but at different times, all other isolates were recovered from animals at different farms.

We recovered isolates from different clinical backgrounds: 8 from skin infections, 4 from genital tract infections, and 1 each from respiratory infections, mastitis, and otitis. We collected samples from skin and ear infections with sterile cotton swabs and collected the milk sample from the mastitis case aseptically in a sterile tube. Rabbits with genital tract or lung infections were euthanized, at farms or laboratories, and necropsied under aseptic conditions; clinical specimens were collected with forceps and scissors scrubbed in 70% ethanol. Samples taken at farms were transported to the laboratory in refrigerated polyethylene bags and processed within 24 hours after sampling. 

Clinical specimens were sampled onto blood agar plates that were incubated at 37°C for 24–48 hours. Identification of isolates as *S. pyogenes* was based on colony morphology, β-hemolysis, and biochemical characteristics using the commercial identification system rapid ID 32 STREP (BioMerieux, Marcy L’Étoile, France). Biochemical identification was also confirmed by sequencing the 16S rRNA gene (*8*).

### Antimicrobial Drug Susceptibility Tests

We performed drug susceptibility testing using the Clinical and Laboratory Standards Institute broth microdilution method (*9*) in Mueller–Hinton broth supplemented with 5% lysed horse blood. We determined the susceptibilities of the isolates with a commercially available susceptibility test (CMV3AGPF Sensititer standard panel; Trek Diagnostics, West Essex, UK) performed according to the manufacturer’s instructions. The agents we tested were penicillin (0.25–16 μg/mL), erythromycin (0.25–8 μg/mL), vancomycin (0.25–32 μg/mL), daptomycin (0.25–16 μg/mL), chloramphenicol (2–32 μg/mL), linezolid (0.5–8 μg/mL), tetracycline (1–32 μg/mL), quinupristin (0.5–32 μg/mL), tigecycline (0.05–0,5 μg/mL), streptomycin (512–2048 μg/mL), kanamycin (128–1024 μg/mL), lincomycin (1–8 μg/mL), and gentamicin (128–1024 μg/mL). In addition, we determined MICs of clindamycin, erythromycin, and tetracycline by Etest (AB Biodisk, Solna, Sweden). We interpreted the results using the Clinical and Laboratory Standards Institute breakpoints for streptococci (*9*) for penicillin, erythromycin, vancomycin, daptomycin, chloramphenicol, tetracycline, and quinupristin; the European Committee on Antimicrobial Susceptibility Testing breakpoints for tigecycline and linezolid (http://www.eucast.org/clinical_breakpoints); and the Comité de l’Antibiogramme de la Société Française de Microbiologie breakpoints (*10*) for streptomycin, kanamycin, lincomycin, and gentamicin.

### Macrolide Resistance Phenotype

To identify macrolide resistance phenotypes, we used a double-disk diffusion test (D-zone test) using erythromycin (15 μg) and clindamycin (2 μg) disks, as described by Hasenbein et al. (*11*). Isolates with blunting of the clindamycin inhibition zone around the disk adjacent to the erythromycin disk were considered to have an iMLS_B_ phenotype (erythromycin resistant and clindamycin inducible). Clindamycin-susceptible isolates without blunting indicated an M phenotype (erythromycin resistant and clindamycin susceptible). Isolates that were resistant to both antimicrobial drugs were considered to have a cMLS_B_ phenotype (constitutive erythromycin and clindamycin resistant).

### Detection of Macrolides and Tetracycline Resistance Genes

We extracted DNA according to the protocol in the US Centers for Disease Control and Prevention (CDC) *S.* *pyogenes* sequence database (http://www.cdc.gov/ncidod/biotech/strep/protocols.htm). We screened all erythromycin-resistant isolates by PCR for the erythromycin resistance genes *erm*B (*12*), *erm*A (*13*), *mef*A (*14*), and *msr*D (*15*). We tested tetracycline-resistant isolates for the tetracycline resistance genes *tet*M and *tet*O (*14*).

### Detection of Virulence Genes

We tested the *S. pyogenes* isolates for the presence of the virulence genes *spe*A*, spe*B, *spe*C, *spe*F, *spe*G, *spe*H, *spe*J, *spe*M, *ssa*, and *sme*Z by PCR. We used primers and conditions described previously (*16**,**17*).

### PFGE Analysis, MLST, and *emm* Typing

For PFGE analysis, genomic DNAs of the *S. pyogenes* isolates were prepared and digested with *Sma*I restriction enzyme (MBI Fermentas, Vilnius, Lithuania) following a previously published protocol (*18*). We performed MLST following the method established by Enright et al. (*19*) and assigned the allele and sequence type (ST) according to the PubMLST website (http://pubmlst.org/spyogenes). We amplified and sequenced the *emm* gene according to the protocol of the CDC International Streptococcal Reference Laboratory (http://www.cdc.gov/streplab/protocol-emm-type.html). We compared the sequences of the *emm* genes with those in the CDC database using BLAST analysis (http://www.cdc.gov/ncidod/biotech/strep/strepblast.htm) for type assignment.

## Results

We observed 2 *emm* types (Table 2): *emm*12 was the most frequent (11 isolates), followed by *emm*77 (4 isolates). Two pulsotypes (A and B) were generated after typing the isolates by PFGE with the restriction enzyme *Sma*I; 11 isolates were pulsotype A and 4 isolates pulsotype B (Figure). Similarly, we observed 2 genetic linages (ST26 and ST63) after MLST analysis.

**Table 2:** Testing results for the 15 isolates characterized in study of *Streptococcus pyogenes* from animal specimens, Spain*

Isolate	*emm *type	PFGE profile	MLST type	MIC, mg/L				Macrolide resistance		TET resistance genes	Virulence genes
ERY	CLIN	TET	Phenotype	Genotype
M50163	12	A	ST36	>256	32	96		cMLS_B_	*mef*A/e*rm*B	*tet*M*/tet*O	*spe*B/*spe*F/*spe*G
M79144	12	A	ST36	>256	0.75	48		M	*mef*A/e*rm*B	*tet*M*/tet*O	*spe*B/*spe*F/*spe*G
M78761	12	A	ST36	>256	0.75	32		M	*mef*A/e*rm*B	*tet*M*/tet*O	*spe*B/*spe*F/*spe*G
M75791	12	A	ST36	6	0.09	32		M	*mef*A/e*rm*B	*tet*M*/tet*O	*spe*B*/spe*F*/spe*G
M75539	12	A	ST36	8	0.19	24		M	*mef*A/e*rm*B	*tetM/tetO*	*spe*B*/spe*F*/spe*G
M75533	12	A	ST36	16	0.19	32		M	*mef*A/e*rm*B	*tet*M*/tet*O	*spe*B*/spe*F*/spe*G
M75123	12	A	ST36	12	0.19	48		M	e*rm*B	*tet*M*/tet*O	*spe*B*/spe*F*/spe*G
M73512	12	A	ST36	0.25	0.12	32				*tet*M*/tet*O	*spe*B*/spe*F*/spe*G
M72636	12	A	ST36	>256	>256	48		cMLS_B_	e*rm*B	*tet*M*/tet*O	*spe*B*/spe*F*/spe*G
M72193	12	A	ST36	>256	1.5	96		iMLS_B_	e*rm*B	*tet*M*/tet*O	*spe*B*/spe*F*/spe*G
83639	12	A	ST36	>256	0.38	48		M	e*rm*B	*tet*M	*spe*B*/spe*F*/spe*G
83553	77	B	ST63	0.19	0.12	64					*spe*B/*spe*F/*spec*
85374	77	B	ST63	0.12	0.09	64				*tet*O	*spe*B/*spe*F/*spec*
M75768	77	B	ST63	0.12	0.09	32				*tet*M*/tet*O	*spe*B/*spe*F/*spe*C
M82209	77	B	ST63	0.19	0.12	64				*tet*O	*spe*B/ *spe*F/*spe*C

*ERY, erythromycin; CLIN, clindamycin; MLST, multilocus sequence typing; PFGE, pulsed-field gel electrophoresis; ST, sequence type; TET, tetracycline.

**Figure:**
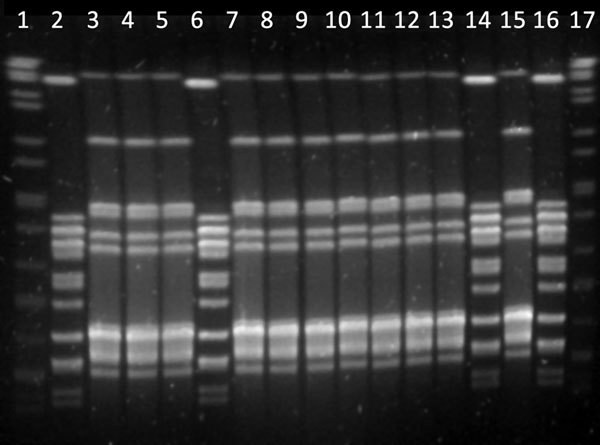
Pulsed-field gel electrophoresis patterns of *Sma*I-digested DNA of clinical isolates of *Streptococcus pyogenes* from animal specimens, Spain, 2006–2014. Lanes 1 and 17, DNA molecular size marker; lanes 3, 4, 5, 7, 8, 9, 10, 11, 12, 13, 15, and 16, isolates M50163, M79144, M78761, M75791, M75539, M75533, M75123, M73512, M72636, M72193, and M83639, respectively (pulsotype A); lanes 2, 6, 14, and 16, isolates 83553, 85374, M75768, and M82209, respectively (pulsotype B).

All 15 *S. pyogenes* isolates were susceptible to penicillin (MIC <0.25 mg/L), vancomycin (MICs <0.25 to 0.5 mg/L), daptomycin (MIC <0.25 mg/L), chloramphenicol (MICs <2 to 4 mg/L), tigecycline (MICs <0.015 to 0.12 mg/L), and gentamicin (MIC <128 mg/L). Additionally, all isolates but 1 were susceptible to kanamycin (MIC <128 mg/L), and 12 isolates showed susceptibility to linezolid (MICs <2 mg/L), streptomycin (MICs >2,048 mg/L), and lincomycin (>8 mg/L). On the other hand, all isolates were resistant to tetracycline, with MICs ranging from 24 to 96 mg/L using Etest (Table 2). Eleven isolates showed tetracycline-resistant genotype *tet*M*/tet*O, 2 isolates *tet*O, and 1 isolate *tet*M (Table 2).

Most isolates (7/15) exhibited the M phenotype, 2 isolates the phenotype cMLS_B_, and 1 the phenotype iMLS_B_ (Table 2). The macrolide-resistant genotype *mef*A/*erm*B was the most frequently observed, seen in all isolates but 1 with the M phenotype and in the isolate with phenotype cMLS_B_. The genotype *erm*B was observed alone in 1 isolate of each phenotype. No isolate carried the *msr*D or *erm*A macrolide-resistant determinants.

We detected the chromosomal-encoded *spe*B and *spe*F genes in all isolates. We observed 2 different virulence gene profiles based on the presence/absence of the *spe*G and *spe*C genes. We detected the genotype *spe*G in 11 isolates and the genotype *spe*C in 4 isolates (Table 2).

We grouped the 15 *S. pyogenes* isolates into 2 different clones on the basis of *emm*-MLST-PFGE-virulence genes profile combinations. Clone 1 grouped isolates characterized by the combined genotype *emm*12-ST36-pulsotype A-*spe*B/*spe*F/*spe*G, whereas isolates of clone 2 were characterized by the genotype *emm*77-ST63-pulsotype B-*spe*B/*spe*F/*spe*C (Table 2). In addition, isolates of clone 1 were erythromycin resistant, mainly exhibiting an M phenotype, and isolates of clone 2 were erythromycin susceptible.

## Discussion

*S. pyogenes* is a human pathogen that has rarely been isolated from animals. It has been isolated from abscesses in cervical and mesenteric lymph nodes and liver of a free-living European hedgehog (*E. europaeus*) and from 2 dogs with severe colonic disease and conjunctivitis (*5**–**7*). Here we describe the detailed characterization of animal *S. pyogenes* isolates from different clinical specimens obtained from rabbits (n = 14) and sheep (n = 1) in Spain during 2006–2014. This pathogen was recovered mainly from noninvasive cases, with skin infections being the most common clinical presentation (n = 6), followed by genital tract infections (n = 4) (Table 1). *S. pyogenes* was isolated from all skin clinical samples together with *Staphylococcus aureus*, a well-recognized pathogen associated with different skin diseases in animals (*20*). These results indicate that although *S. pyogenes* should be able to colonize the skin of animals, it is difficult to ascertain its etiologic significance in skin infections. However, *S. pyogenes* was isolated in pure culture from clinical specimens of the genital tract, ears, mammary glands, and lungs in rabbits, indicating the potential role of *S. pyogenes* in these infections.

Most of the *S. pyogenes* isolates we tested (n = 11) exhibited the genotype *emm*12-ST36, which has been isolated repeatedly from humans in different countries (*21**–**27*), including Spain (*28**–**30*). This genotype can exhibit an M phenotype (*31*) and has been associated with skin and soft tissue infections (*32*), data that fit with our results, as more than half of the isolates with this genotype were isolated from abscesses and dermatitis (Table 2). The genotype *emm*77-ST63 that we identified in 4 animal isolates has also been detected in human *S. pyogenes* isolates (*21**,**25**,**33*), but unlike human isolates, the isolates in our study were erythromycin and clindamycin susceptible (Table 2). 

All 11 isolates in clone 1 (pulsotype A) exhibited PFGE profiles that were indistinguishable from each other, and all 4 isolates in clone 2 also exhibited PFGE profiles that were indistinguishable PGFE from each other (pulsotype B; Figure). Isolates of *S. pyogenes* usually exhibit high levels of genetic diversity (*4*). Thus, the fact that we identified only 2 clones in different isolates collected over a period of 8 years was unexpected. The possibility of a common source of infection is very unlikely because all isolates were recovered at different times from different animals in farms located at geographically distant locations spread throughout Spain, without any epidemiologic relationship (Table 1). In addition, clinical specimens were processed independently in the same laboratory by highly qualified and trained personnel, which makes the possibility of a cross-contamination in the laboratory unlikely. 

Under these conditions, multiple human-to-animal transmission events should be the most likely origin of these genotypes in sheep and rabbits. Another possible explanation could be that genotypes ST36 and ST63, although originating from humans, represent genetic linages with a specific host tropism, mainly for rabbits, which contributed to their successful dissemination in these animals, as observed with other streptococci (*34*). Cases of *S. pyogenes* infection were not recorded among the personnel working in the rabbit farms and sheep flock from which *S. pyogenes* was isolated. Asymptomatic human carriers have a key role in *S. pyogenes* transmission (*35*). For these reasons and even though screenings to identify asymptomatic *S. pyogenes* carriers were not carried out, we can speculate that asymptomatic employees were the most probable source of *S. pyogenes* in the animals included in the study. Although we cannot infer from the results of this study that animals, mainly rabbits, may represent a new reservoir of *S. pyogenes*, the results clearly indicate the ability of human-derived *S. pyogenes* isolates to colonize and infect animals, which could be more frequent than has been recognized until now.

Isolates with the genotype *mef*A/e*rm*B usually correlate with the cMLS_B_ phenotype, but 5 of the 6 *S. pyogenes* isolates with the *mef*A/*erm*B genotype in our study exhibited M phenotype (Table 2), which agrees with previous observations (*29*). The *erm* gene usually confers co-resistance to macrolides, lincosamides, and streptogramins. Curiously, all M phenotype isolates in our study showed susceptibility to clindamycin and were positive for the *emr*B gene. This result, although unusual, has also been observed previously in *S. pyogenes* isolates from different countries (*26**,**36**–**38*). A possible explanation could be that the *erm*B gene was nonfunctional in the isolates with clindamycin-susceptible phenotypes. The isolate M72193 exhibited the iMLS_B_ phenotype but was *erm*A-negative (Table 2). This result, although infrequent, has also been observed in previous studies (*39*). Isolates with the iMLS_B_ phenotype have been further subdivided into 3 distinct types: type A, associated with the presence of the *erm*B gene; and types B and C, associated with the presence of the *erm*A gene (*40**,**41*). This isolate carried the *erm*B gene (Table 2), suggesting therefore an iMLS_B_-A phenotype. 

Unlike most human *S. pyogenes* isolates, which usually carry either *tet*M or *tet*O genes, most of the isolates in this study (n = 11) carried both genes (Table 2). Human isolates with the combination of *tet*M and *tet*O tetracycline-resistance genes have been identified previously in Spain (*29*). Another uncommon result was the identification of 1 isolate (83553) that was resistant to tetracycline (MIC 64 mg/L) but lacked resistance *tet*M and *tet*O genes (Table 2) commonly associated with tetracycline resistance in *S. pyogenes* (*42*). However, tetracycline-resistant strains and negativity to these genes have also been reported (*43*). Further studies will be necessary to elucidate the precise mechanism of resistance to tetracycline in this strain.

In summary, this study provides a detailed characterization of animal *S. pyogenes* isolates associated with different clinical backgrounds. This pathogen should be considered by veterinary microbiologists when processing clinical material from animals.
